# Level of self-care practice among diabetic patients in Ethiopia: a systematic review and meta-analysis

**DOI:** 10.1186/s12889-020-8425-2

**Published:** 2020-03-12

**Authors:** Daniel Bekele Ketema, Cheru Tesema Leshargie, Getiye Dejenu Kibret, Moges Agazhe Assemie, Alehegn Aderaw Alamneh, Getachew Mullu Kassa, Animut Alebel

**Affiliations:** 1 Department of Public Health, College of Health Sciences, Debre Markos University, P.O. Box 269, Debre Markos, Ethiopia; 2 Department of Nutrition and Food Science, College of Health Sciences, Debre Markos University, Debre Markos, Ethiopia; 3 Department of Nursing, College of Health Sciences, Debre Markos University, Debre Markos, Ethiopia

**Keywords:** Diabetes, Self-care, Blood glucose monitoring, Systematic review, Meta-analysis

## Abstract

**Background:**

Diabetes Mellitus (DM) is increasingly become a serious global public health concern in developed and developing countries including Ethiopia. It imposes significant burden of care on the individual, health care professionals and health system. As the result, immense need of self-care behaviors in multiple domains like food choices, physical activity, foot care, and blood glucose monitoring is required. However, there is no national study on diabetic self-care practices in Ethiopia. This meta-analysis, therefore, aims to estimate the pooled level of self-care practice among individuals living with diabetes mellitus in Ethiopia.

**Methods:**

The systematic review was reported according to the Preferred Reporting Items for Systematic Reviews and Meta-Analysis (PRISMA) guideline. We systematically searched the databases: PubMed /MEDLINE, EMBASE, Google Scholar, and Science Direct for studies conducted in Ethiopia about self-care practice of diabetes patients. We have included all cross-sectional studies, which were published until August 20th,2019. Data were analyzed using _STATA_™ version 14.1 software, and the pooled prevalence with 95% confidence intervals (CI) were presented using tables and forest plots. The presence of statistical heterogeneity within the included studies was evaluated using I-squared statistic. We used Higgins and Egger’s test to identify evidence of publication bias. The random-effects meta-analysis model was employed to estimate the pooled proportion of good diabetic self-care practices.

**Results:**

We included 35 studies (with 11,103 participants) in this meta-analysis. The overall pooled prevalence of good diabetes self-care behavior among diabetic patients was 49% (95% CI:43, 56%). When categorized by the major domains of diabetes self-care, the pooled estimate of dietary practice was 50% (95% CI:42, 58%), for self- monitoring of blood glucose was 28% (95% CI:19, 37%), for recommended physical activity was 49% (95% CI:38, 59%), and for diabetic foot-care was 58% (95% CI: 41, 74%).

**Conclusion:**

More than half of diabetic patients in Ethiopia had poor diabetes self-care practice. High percentage of diabetic patients also had poor dietary practice, self- monitoring of blood glucose, physical activity, and diabetic foot care. Therefore, intervention programs should focus on improving the knowledge level of diabetic patients to improve the self-care practice of diabetic patients.

## Background

Diabetes Mellitus (DM) related clinical complications are most important causes of morbidity and mortality, and have considerable effect on the patients’ quality of life and productivity [1, 2]. Globally, approximately half a billion people live with diabetes, and almost 80% of diabetes burden was shared by low and middle income countries including Ethiopia [3, 4]. According to the International Diabetes Federation (IDF) report, there were 2,567,900 cases of diabetes in Ethiopia making the adult prevalence of 5.2% [3]. Due to the rapid expansion of urbanization, unhealthy diets, and sedentary change of life style, the incidence of DM keeps increasing [3, 5].. The chronic nature of DM causes significant personal suffering and economical difficulty in the families [3, 4, 6, 7]. Moreover, DM have not impose only personal suffering; due to its continued needs and demands of individual effort, it was considered as a major challenge for health care workers as well [8].

In order to reduce the burden posed to health systems and affected individuals, patients with diabetes need to adopt certain diabetes self-care behaviors. Diabetes self-care behavior is defined as an evolutionary process of development of knowledge or awareness by learning to survive with the complex nature of the diabetes in a social context [4, 6, 9]. American Diabetes Association (ADA) has set a list of essential self-care activities, which used for monitoring blood glucose at recommended level, prevent diabetic complications and improving the quality of life of diabetic patients [6, 7]. These diabetes self-care includes: self-monitoring of blood glucose (SMBG), nutrition, physical activity and diabetic foot care [1, 6, 10]. Diabetes is a complex, chronic illness requiring continuous medical care with multifactorial risk-reduction strategies beyond glycemic control [3]. Several studies reported that, ongoing patient self-care education and support are critical to preventing acute complications and reducing the risk of long-term complications [3, 11, 12].

DM self-care activities are owned by patients and their families;, as a result, there should be consistent and effective measure for diabetes self-care through dietary and lifestyle modification complemented with supportive role of health care providers [4, 13–15]. Self-monitoring of glycemic combined with regular physical activity are considered as a cornerstone of diabetes cares to ensure patient participation in achieving and maintaining at recommended level of blood glucose [6, 16–19].

The majority of patients with diabetes can significantly reduce the chance of developing long-term complication by improving self–care activities. To date, the level of DM self- care practice have been reported in several studies in Ethiopia, which ranges from 22.5 to 76.8% [1, 20–35]. However, most of these studies have offered district level information. As a result of variability of findings across previously existing studies, producing pooled proportion of DM self-care at the national level is needed. Moreover, comprehensive estimates of the extent of DM self-care levels are needed for the programmatic management of diabetes within the context of diabetes care. Therefore, this systematic review and meta-analysis was undertaken to produce pooled estimates of DM self-care levels in Ethiopia.

## Methods

### Data source and search strategy

Published and unpublished research articles that were conducted to assess a minimum of one variety of diabetic self-care (overall diabetic self-care, diet, physical activity, self-monitoring of glucose, foot care) in Ethiopia were included during this study. An intensive search was done from PubMed/MEDLINE, and EMBASE online databases to access articles done on the diabetes self-care practices. Moreover, Google Scholar and Science direct were accustomed to retrieve articles. Besides, reference lists of screened studies were checked. The search was administered by two authors (DBK and AA) independently. The term ‘diabetes’ was searched with all of the subsequent terms as a mix of free text and thesaurus terms in numerous variations: diabetes self-care, dietary care, foot care, physical activity, glycemic control, and Ethiopia. Moreover, the subsequent keywords were accustomed to retrieve studies from PubMed database; (((((Diabetes self-care) OR diabetes dietary care) OR diabetes foot care) OR diabetes physical activity) OR self-monitoring of blood glucose) AND Ethiopia. Studies that were relevant by title and abstract were assessed by full text to see those that provided adequate data to be included in our meta-analysis. This systematic review and meta-analysis was reported per preferred reporting items for Systematic Review and Meta-Analysis (PRISMA) guideline [36].

### Inclusion criteria

- **Study setting**: Studies done in Ethiopia.
- **Study participants:** Studies conducted among all Diabetes Mellitus patients.
- **Publication status:** All published and unpublished articles.
- **Language:** Only studies published in the English were included
- **Types of studies:** Studies that employed observational study design.
- **Publication date:** The authors included articles published until August 20,2019

### Exclusion criteria

- Despite the above-mentioned preset eligibility criteria, articles which we were unable to access the full-texts after two email contacts of the principal investigator of the particular study were excluded from the final analysis.

### Outcome measures

Diabetes self-care was defined as good self-care practice and poor self-care practice [24, 25]. In this meta-analysis, we measure diabetes self-care practice using four domains: dietary practices, engagement in regular exercise, foot-care and blood glucose monitoring [3, 6].

### Dietary practice

Patients who scored above the mean value for the response were classified as having good dietary practice and poor dietary practice otherwise.

### Glycemic control

The level of glycemic control was indicated as ‘good glycemic control’ when FBS results were between 70 and 130 mg/dL (i.e. an average of three measures at different visits).

### Regular exercise

20–30 min of aerobic exercise such as walking or swimming 3–4 days per week [37].

### Foot care practice

a total practice score of ≥50% of maximum score is categorized as good foot care practice, while a total practice less than a score of 50% categorized as poor foot care practice [38].

### Data extraction and quality assessment

Seven authors (DBK, AAA, GDK, AA, AAA, and CTL) done data abstraction using pre-piloted data extraction format prepared in Microsoft™ Excel spreadsheet. The seventh author (GMK) reconciled any disagreements among the six authors. From each study, we have extracted data on the study location, region, publication year, study design, sample size, and first author name for the overall diabetes self-care and for each domains of diabetes self-care practice (Supplementary File 1, Supplementary File 2, Supplementary File 3, Supplementary File 4, and Supplementary File 5).

The quality of included studies has been assessed in accordance with the Newcastle-Ottawa Quality assessment scale [39]. Two independent reviewers (DBK & GDK) done a full-text quality assessment. Disagreement between the two reviewers was found to be very low (1.5%) and they resolved this by discussion. Then, a combined quality score ranging from 0 to 10 was assigned to each included articles.

### Heterogeneity and publication bias

The presence of statistical heterogeneity within the included articles was assessed using I-squared statistic. Accordingly, heterogeneity was classified as low, moderate, or high when the value of I-squared were 25, 50, and 75%, respectively [40]. Begg and Egger weighted regression method were employed to detect evidence of publication bias. *P*-value < 0.05 was considered as presence of significant publication bias [41].

### Statistical analysis

Relevant data from every primary study was extracted using a Microsoft Excel format. Data were then exported to STATA™ Version fourteen software package for analysis. The pooled estimate was computed using metan SATA command. Results were presented using tables and forest plots with 95% confidence intervals (CI). We have used Higgins and Egger’s test to identify publication bias. As a result of high level of heterogeneity among included studies, random-effects model was used to produce Der Simonian and Laird’s pooled estimate. Additional advanced statistical analyses like univariate meta-regression and sensitivity analysis were performed to identify the potential sources of heterogeneity and to assess the influence of a single study on the overall pooled estimate respectively.

## Results

### Selection and identification of studies

**Fig. 1:**
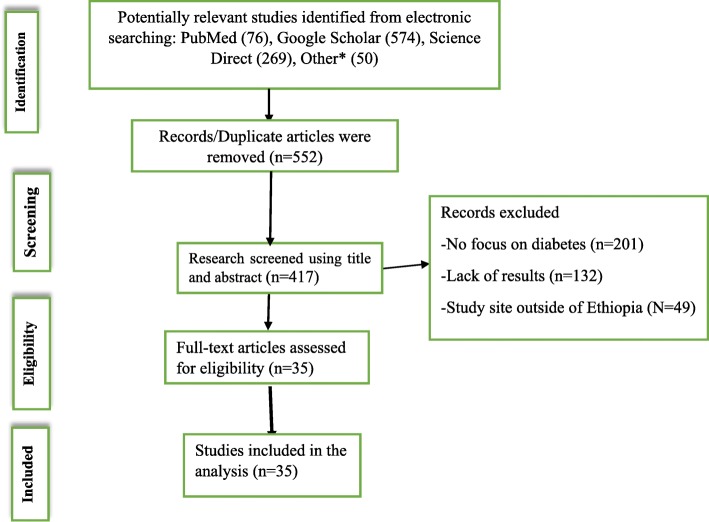
PRISMA flow diagram which shows the studies selection of the meta-analysis on levels of diabetes self-care practice in Ethiopia

A total of 969 articles were retrieved from electronic databases and other sources. Title and abstracts were screened and duplicated or irrelevant articles were removed using EndNote × 7. Accordingly, 552 duplicate articles were removed. From the remaining 417articles, 382 articles were excluded because their titles and abstracts were not in-line with our inclusion criteria (not report the outcome of the interest, studies conducted outside of Ethiopia). Finally, a total of 35 articles were included for this systematic review and meta-analysis. The detailed selection procedures were described in Fig. 1.

### Description of included studies

We included 35 cross-sectional studies (with 11,103 participants) in the final systematic review and meta-analysis. The smallest sample size was 102 obtained from a study conducted at Ambo Hospital Oromia region of Ethiopia [35]. Whereas, the largest sample size was 419 reported from a study at Tikur Anbessa Specialized Hospital, Addis Ababa, Ethiopia [42]. The detail characteristics of included studies was presented in Table 1.

**Table 1:** Study characteristics of included articles for the final systematic review and meta-analysis on diabetes self-care among diabetic patients in Ethiopia

No.	Author	Year	Region	Sample size	Reported outcome percentage (95% CI)					Quality assessment score
					Overall self-care	Dietary-practice	Physical-activity	SMBG	Foot-care	
1	Berhe, K, et al. [23].	2017	Tigray	310	49.4 (43.8, 54.9)	32.9(27.7, 38.1)	74 (69.1, 78.8)	14 (10.1, 17.8)	51.3 (45.7, 56.8)	7
2	Kassahun CW. et al. [2]	2017	Oromia	284	56.6 (31.5, 81.7)	–	**–**	–	–	8
3	Chali, S.W. et al. [24]	2018	Benishangul	383	54.3 (49.3, 59.3)	–	**–**	–	–	6
4	Worku, A, et al. [43].	2015	Addis Ababa	403	–	48.6 (43.7, 53.5)	**–**	41.2 (36.4, 46.0)	–	4
5	Demilew YM. et al. [44]	2018	Amhara	401	–	35.9 (31.2, 40.6)	**–**	–	–	8
6	Tiruneh SA.., et al. [34]	2019	Amhara	385	63.1 (58.8, 67.9)	–	**–**	–	–	5
7	Gesesew H. et al. [28]	2016	Oromia	309	**–**	–	49.2(43.6, 54.8)	71 (65.0, 76.0)	–	7
8	Hailu, E., et al. [37]	2012	Oromia	343	**–**	55.6 (50.3, 60.8)	64.9 (59.8, 69.9)	2.6 (0.91, 4.3)	82.9(78.9, 86.8)	9
9	Niguse H. et al. [32]	2016	Tigray	338	25.5 (20.8, 30.1)	–	–	–	**–**	6
10	Ayele K. et al. [1]	2012	Harai	222	39.2 (32.8, 45.8)	55.7 (51.2, 64.2)	31.1 (25.0, 37.2)	2.3 (0.32, 4.3)	**–**	7
11	Feleke SA. et al. [27]	2013	Amhara	410	36.8 (32.1, 41.5)	57.1 (52.3, 61.9)	–	23.6 (19.5, 27.7)	**–**	9
12	Addisu,Y, et al. [21]	2014	SNNP	310	76.8 (72.1, 81.5)	49.7 (44.1, 55.3)	44.5 (38.9, 50.0)	20.0 (15.5, 24.4)	**–**	5
13	Bonger, Z, et al. [45]	2018	Addis Ababa	419	**–**	24.1 (20.0, 28.2)	46.3 (41.5, 51.0)	16.5 (12.9, 20.0)	**–**	8
14	Kassahun, T., et al. [29]	2016	Oromia	309	49.2 (43.6, 54.8)	–	–	29.1 (24.0, 34.2)	–	6
15	Aklilu, T., et a**l** [4]	2014	Amhara	303	**–**	80.9 (76.5, 85.3)	45.9 (40.3, 51.5)	20.1 (15.6, 24.6)	80.2 (75.7, 84.7)	4
16	Mariye T, et al. [31]	2018	Tigray	284	37.3 (31.7, 42.9)	–	–	–	–	7
17	Dedefo. G., et al. [25]	2019	Oromia	252	60.7 (54.6, 66.7)	69.4 (63.7, 75.1)	63.5 (57.5, 69.4)	40.5 (34.4, 46.5)	82.9 (78.2, 87.5)	5
18	Mariam. G., et al. [46]	2017	Amhara	279	**–**	97.8 (96.0, 99.5)	81.7 (77.2, 86.2)	63.4 (57.7, 69.1)	36.6 (30.9, 42.2)	9
19	Amente, T., et al. [22]	2014	Oromia	254	55 (48.9, 61.1)	–	–	–	–	6
20	Abate, T.W, et al. [20]	2018	Amhara	416	28.4 (24.0, 32.7)	–	–	–	–	5
21	Deribe, B. et al. [26]	2014	SNNP	216	55.6 (49.7, 61.4)	–	–	–	55.1 (48.5, 61.7)	4
22	Gebrekirstos K. et al. [47]	2015	Tigray	319	**–**	–	–	–	19.3 (14.2, 24.4)	7
23	Sorato, M. et al. [33]	2016	SNNP	194	41.2 (34.3, 48.1)	82.5 (77.1, 87.8)	50.5 (43.5, 57.5)	5.7 (2.4, 8.9)	**–**	8
24	Gurmu, Y. et al. [48]	2018	Oromia	257	54.5 (48.4, 60.6)	–		–	**–**	9
25	Woldu, M., et al. [35]	2014	Oromia	102	–	58.8 (49.2, 68.3)	50 (40.3, 59.7)	83.3 (76.0, 90.5)	**–**	6
26	Tekalegn, Y., et al. [42]	2018	Addis Ababa	412	–	42.5 (37.7, 47.3)	54.4 (49.6, 59.2)	15 (11.5, 18.4)	**–**	5
27	Tegegne GT, et al. [49]	2015	Oromia	111	–	**–**	18 (10.8, 25.1)	23.4 (15.5,31.3)	**–**	8
28	Tamirat A, et al. [50]	2014	Oromia	319	–	**–**	11.9 (8.3, 15.4)	–	**–**	8
29	Wabe T, et al. [51]	2011	Oromia	384	–	**–**	–	41.9 (36.9, 46.8)	**–**	4
30	Angamo T, et al. [52]	2013	Oromia	284	–	**–**	–	18.3 (13.8, 22.8)	**–**	8
31	Yigezu DM. et al. [17]	2017	Oromia	174	–	**–**	–	40.8 (33.5, 48.1)	**–**	5
32	Fasil A. et al. [16]	2019	Amhara	367	–	**–**	–	39.5 (34.5, 44.5)	**–**	6
33	Seid A. et al. [38]	2014	Amhara	313	–	**–**	–	–	53 (47.5, 58.0)	8
34	Mamo M. et al. [30]	2016	Addis Ababa	646	60.3 (56.5,64.0)	**–**	–	–	–	9
35	Abebe SM. et al. [53]	2015	Amhara	391	–	**–**	–	35.3 (30.5, 40.0)	–	4

*SMBG* Self-Monitoring of Blood Glucose

The dash-line (−-) indicate a research not reported particular outcome

*SNNP* South Nation Nationality of people

*CI* Confidence Interval

In the current meta-analysis, seven regions and one administrative town in the country were represented. Most studies took place in Oromia Region (*n* = 13) [2, 17, 22, 25, 28, 29, 35, 37, 48, 49, 51, 52] and Amhara Region (*n* = 9) [4, 16, 20, 27, 34, 38, 44, 46, 53]; followed by Tigray Region (*n* = 4) [23, 31, 32, 47], South Nation Nationality of People (SNNP), *n* = 3)) [21, 26, 33]; Addis Ababa (n = 4) [30, 42, 45, 54]; Benishangul Gumuz region [24], and Harari region [1] (*n* = 1 each). Most studies dealt with self-monitoring of blood glucose (SMBG) (*n* = 21), followed by overall diabetes self-care (*n* = 16), assessment of dietary practice (*n* = 15), and engagement in physical activity (*n* = 14). Only eight studies assessed diabetic foot-care. Quality assessment using the Newcastle-Ottawa Scale for cross-sectional studies showed a quality score ranging from 4 to 9 (Table 1).

### Diabetes self-care practices

#### Overall diabetes self-care practice

**Fig. 2:**
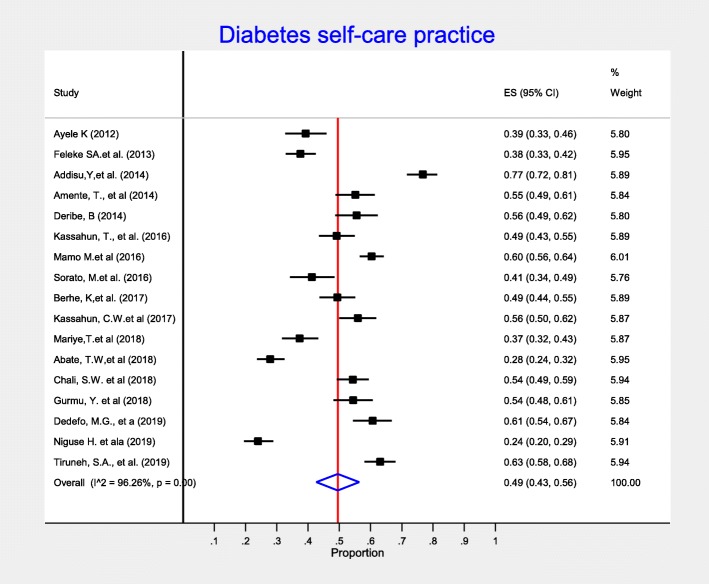
Meta-analysis (forest plot) of the proportion of overall good self-care practice among diabetic patients in Ethiopia

From a total of 35 cross-sectional studies, seventeen studies provided information on overall diabetes self-care practices. As described in the forest plot (Fig. 2), the pooled estimate of the overall diabetes self-care practice was 49% (95% CI:43,56%). High heterogeneity was observed (I-squared = 96.09%), but there was no evidence of publication bias using Egger’s test (*p*-value =0.229). To minimize the potential random variations between the included studies, we performed a sub-group analysis by taking regions of the country where studies conducted. Accordingly, the highest level of diabetes self-care practice was observed from SNNP 58% (95% CI:55, 78%), followed by Oromia region 55% (95% CI: 51, 59%) (Table 2).

**Table 2:** Subgroup level of overall diabetes self-care practice among diabetic patients in Ethiopia by region, 2019

Variable	Characteristics	Included studies	Sample size	Estimate of overall DM self-care % (95% CI)
Region	Amhara	3	1211	43 (23.,63)
	Oromia	5	1356	55 (55,59)
	SNNPR	3	720	58 (55, 0.78)
	Tigray	3	932	37 (22, 52)
	Benishangul Gumz	1	383	54 (49, 59)
	Harari	1	222	39 (33, 46)
	Addis Ababa	1	646	60 (56, 64)

A meta-regression was performed using publication year and sample size as covariates to explore the possible source of heterogeneity. However, there is no significant evidence that indicate the existence of heterogeneity, which explained by using these variables (Table 3). Furthermore, to identify the effect of single study on the overall pooled estimate, we did a sensitivity analysis using a random effects model. Accordingly, there is no evidence of single study influence on the overall pooled estimate.

**Table 3:** Meta-regression output to explore heterogeneity of the pooled prevalence of diabetes self-care practice among diabetic patients in Ethiopia

Variables	Coefficients	P-value	95% conf. Interval
Publication year	−0.5710	0.732	−4.072 2.930
Sample size	0. 0078	0.819	−0.647 0.080

### Diabetes self-care practices across major domains (dietary practices, physical activity, foot-care and blood glucose monitoring)

**Fig. 3:**
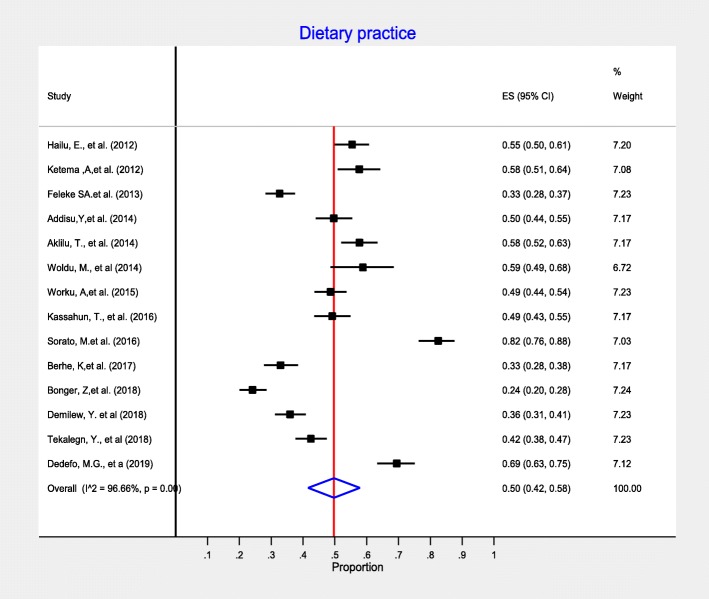
Meta-analysis (forest plot) of the proportion of good dietary self-care practice among diabetic patients in Ethiopia

**Fig. 4:**
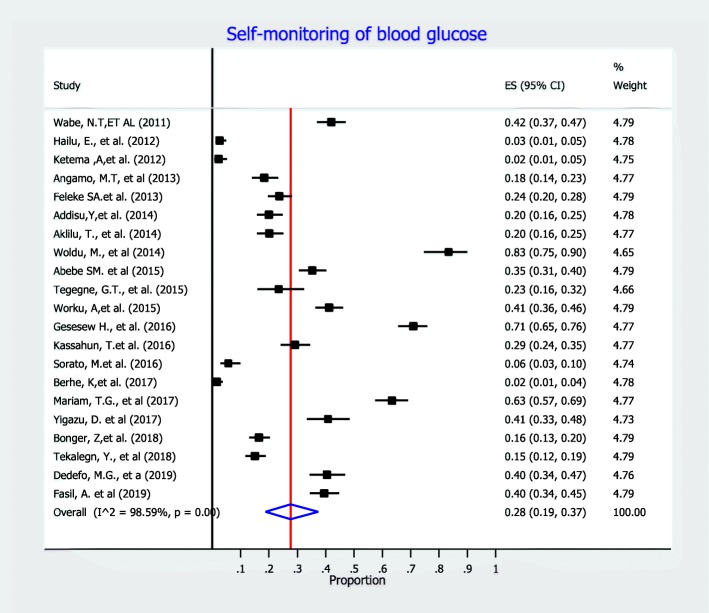
Meta-analysis (forest plot) of the proportion of good practice of self-monitoring of blood glucose among diabetic patients in Ethiopia.

**Fig. 5:**
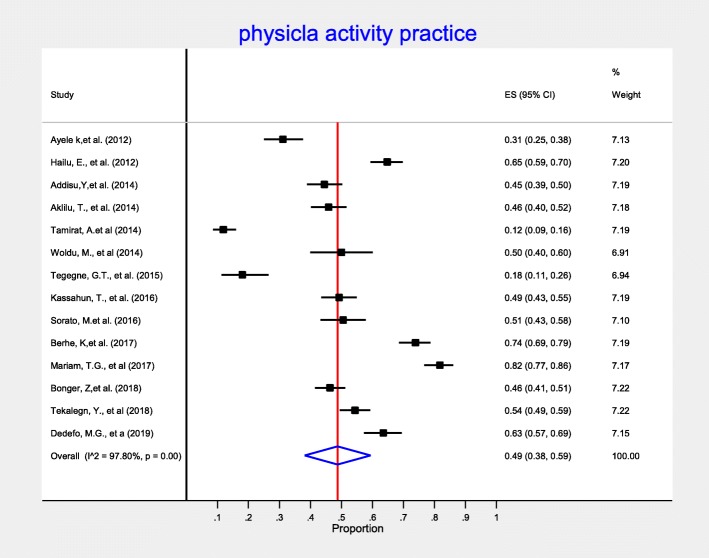
Forest plot of pooled proportion of adequate physical activity among diabetic patients in Ethiopia

**Fig. 6:**
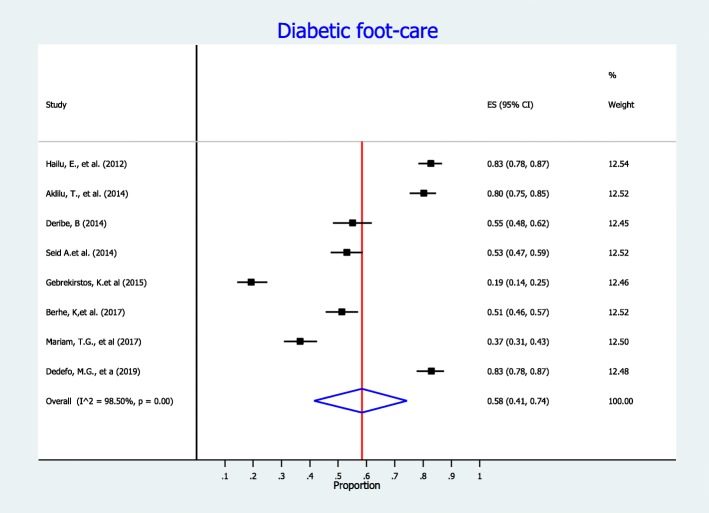
Forest plot of the pooled proportion of good foot-care practice among diabetic patients in Ethiopia

In this meta-analysis, we measure diabetes self-care practice using four domains: dietary practices, engagement in regular exercise, foot-care and blood glucose monitoring. Accordingly, fourteen studies included information on dietary self-care behaviors and reported on patients’ behavior regarding dietary practice. In this meta-analysis, the pooled level estimate of good dietary practice of diabetic patients was 50% (95% CI:42, 58%) (Fig. 3). Concerning on blood glucose monitoring, twenty-one observational studies reported patients’ behavior regarding monitoring of blood glucose. As detailed in Fig. 4, the lowest percentage of diabetic patients that have good glycemic control practice were 28% (95% CI: 19, 37%). That is, 28% of diabetic patients self-monitored and kept their blood glucose levels at recommended level. In addition, fourteen observational studies measured physical activity practice of DM patients. The highest percentage of DM patients who had adequate regular exercise was reported at the University of Gondar Referral Hospital, Amhara region, Ethiopia [46]. As presented in the forest plot (Fig. 5), the pooled level of adequate physical exercise was 49% (95% CI: 38, 59%), which means 49% of diabetic patients had practiced physical activity that met the recommended guidelines. Moreover, eight observational studies reported on the diabetic patients practice on the foot-care. The pooled meta-analysis showed that 58% (95% CI: 41, 74%) of diabetic patients practiced good diabetic foot-care practice (Fig. 6).

## Discussion

This systematic review and meta-analysis was conducted to assess the level of self-care practice among diabetic patients in Ethiopia (including major four domains: like food choices, physical activity, foot care, and blood glucose monitoring). Accordingly, almost half (49%) of diabetic patients in Ethiopia practice the recommended levels of diabetes self-care. This finding was much lower than a study in Nigeria (80%), Thailand (87%), and Iran (74%) [55–57], and higher than the study reported in Kenya (41%) [58]. The difference in culture and economical status, lifestyle difference, and difference in the access to health care facilities and level of education of the general public could be the reasons for the difference. Furthermore, varied methods applied to measure outcome-parameters among countries may be contribute the variation of findings. Diabetes self-care practice demands the involvements of individuals with diabetes and health care providers to increase the adherence of good practice through increasing the knowledge and understanding of the disease and predicting the long-term impacts of the disease. However, difference in the level of education among diabetic patients across the countries, the level of understanding of health care works on diabetes self-care should contribute for the variation diabetes self-care practice across the countries mentioned above.

In this meta-analysis, 50% (95% CI: 42, 58%) of participants have practicing the recommended diet (grains and starchy vegetables, Non-starchy vegetables etc). Similar finding was reported in India (46%) [59]. However, the finding of the current study is higher than the figure reported in Yemen (21%) and Pakistan (17%) [60, 61]. The discrepancy might be explained due to socio-cultural difference, difference in the types of foods available with different nutritional value, as well as level of education provided for diabetic patients. The importance of following a regular dietary plan in terms of both quantity and quality lies in the fact that adequate blood sugar control and proper weight management are linked to it. The World Health Organization (WHO) recommends a minimum of 400 g of fruit and vegetables per day [62]. ADA also recommends 45 to 65% of daily total calorie for patients who are suffering with diabetes [6].

This meta-analysis identify that self-monitoring of blood glucose was a least practice in Ethiopia with pooled prevalence of 28%. This finding was much lower than a report in Alexandria which 64.7% had good compliance of blood glucose control [63]. This low adherence to SMBG practices could probably be attributed to relevant financial barriers to acquisition of glucometer or to undergo a check-up at a health facility [64]. Most patients, who had access to a glucometer at home, checked their glucose level only once a month or at no regular interval [65, 66].

In this meta-analysis almost half (49%) of diabetic patients were engaged in the recommended physical activity. Physical exercise has been considered a cornerstone of diabetes management, along with diet and medication [67]. This finding was almost comparable with a report at USA (39%) [68]. The discrepancy could be explained by the time difference of the research carried out. The evidence reported from USA was conducted in 2007. Whereas almost all included studies which measured physical activity practice of DM patients in this meta-analysis were conducted at the mean year of 2015. Since various initiatives nationally and internationally (Ethiopia Diabetic Associating (EDA), Ministry of Health (MOH)) have been instigated by several organizations between these motioned time period, the proportion of diabetic patients who were engaged in the recommended physical activity could be increased. Researches have shown that physical activity can lower bad cholesterol and raise good cholesterol, improve body’s ability to use insulin [50, 67, 69]. Exercise have also beneficial effect for diabetic patients to reduce the development of cardiovascular complication with regard to glucose control [70].

In this meta-analysis, more than half of diabetes patients have good foot-care practice with pooled prevalence of 58% (95% CI:41, 74%). This finding was comparable with findings from Siri Lanka (53%) and South Africa (69.2%) [71]. However,the finding was much higher than the study reported in Nigeria (10%) [72]. This discrepancy could be explained by diabetic foot care involves many different professionals and the patient presentations also diverse. Successful management of diabetic foot problem needs the expertise of multidisciplinary team with implementation of universal good care. This involves good attitude of healthcare professionals, patients and health-care system.

### Limitation of the study

This systematic review and meta-analysis have certain limitations. First, methods applied to measure outcome-parameters varied among included studies. Second, included studies used self-report as a measure of outcomes, and this may have introduced bias. Multiple methods may be required to detect those who report good practice but who may in fact may have poor practice.

## Conclusion

More than half of diabetic patients had poor diabetes self-care practice. Therefore, health care personnel and Ethiopian Diabetic Association (EDA) should increase the patient’s awareness to the importance of self-care practices domains and strongly promote the practice among diabetes patients by strengthening different local program working on diabetes. Future efforts and resource investments in public health system need to strengthen the distribution of structured diabetes-care programs which need to be adapted to the Ethiopia-context.

## Supplementary information

**Additional File 1.** Over all diabetic self-care data set.

**Additional File 2.** Dietary practice data set.

**Additional File 3.** Self-monitoring blood glucose dataset.

**Additional File 4.** Physical activity data set.

**Additional File 5.** Diabetic foot-care data set.

## Abbreviations

ADA: American diabetes association
CI: Confidence interval
EDA: Ethiopia diabetes association
PRISMA: Preferred reporting items for systematic reviews and meta-analyses
SE: Standard error
SMBG: Self-monitoring of blood glucose
SNNPR: South nation and nationalities people of the region
WHO: World health organization
